# Exploring Hope in Patient–Caregiver Dyads Living with Advanced Chronic Disease in Portugal: A Phenomenological Study

**DOI:** 10.3390/healthcare14182985

**Published:** 2026-09-12

**Authors:** Letícia Figueirinha Crespo, Eduarda Guiné, Sidarth Pernencar, Ana Querido, Feten Fekih-Romdhane, Carlos Laranjeira

**Affiliations:** 1 School of Health Sciences, University of Leiria and Oeste, 2411-901 Leiria, Portugal; 2 Cardiology Department, Hospital of Santo André, Local Health Unit of the Leiria Region, 2410-197 Leiria, Portugal; 3 Centre for Innovative Care and Health Technology (ciTechCare), University of Leiria and Oeste, 2414-016 Leiria, Portugal; 4 RISE Health Research Group, University of Porto, 4200-450 Porto, Portugal; 5 Faculty of Medicine of Tunis, Tunis El Manar University, Tunis 1068, Tunisia; 6 The Tunisian Center of Early Intervention in Psychosis, Department of Psychiatry, Razi Hospital, Tunis 2010, Tunisia; 7 Comprehensive Health Research Centre (CHRC), University of Évora, 7000-801 Évora, Portugal

**Keywords:** hope, palliative care, chronic disease, dyads, phenomenology

## Abstract

**Background/Objectives:** Advanced chronic diseases significantly compromise the quality of life of patients and their family caregivers due to the high symptom burden, psychosocial distress, and increasing care dependency. Despite recommendations for the early integration of these individuals into Palliative Care (PC), understanding hope as a resource for adaptation to the illness trajectory remains limited. This study aimed to describe the lived experience of hope among dyads consisting of individuals with advanced chronic disease and their family caregivers. **Methods:** A qualitative descriptive phenomenological design, grounded in Amedeo Giorgi’s methodology, was used. Data were collected through semi-structured interviews with 13 patient–caregiver dyads living with advanced chronic obstructive pulmonary disease or advanced heart failure. Data were analysed following the procedures of descriptive phenomenological analysis, and the study was reported in accordance with the Standards for Reporting Qualitative Research (SRQR) guidelines. **Results:** The lived experience of hope was organized into four essential constituents: (1) hope as a horizon of possibilities; (2) everyday anchors of hope; (3) challenges to sustaining hope; and (4) cultivating hope in the face of adversity. Findings revealed that hope was experienced as a dynamic, multidimensional, and relational phenomenon. Participants described hope as being repeatedly reshaped in response to changing illness-related circumstances. Dyads identified multiple sources of hope, including family relationships, legacy, spirituality, and short-term goals, alongside challenges such as disease progression, caregiver burden, significant losses, and social isolation. **Conclusions:** Within this sample, hope emerged as a fundamental resource for adaptation to advanced chronic illness, enabling dyads to find meaning in the illness experience, cope with uncertainty, and preserve quality of life. As a phenomenon shared between the patient and the family caregiver, hope highlights the importance of dyad-centred approaches and its integration into palliative care to promote more humanized, relational, and responsive care that addresses the emotional, social, and spiritual needs of families.

## 1. Introduction

Chronic diseases are among the leading causes of mortality in Europe, representing a major challenge for healthcare systems and society [1]. According to the World Health Organization (WHO) [2], chronic diseases are characterized by their long duration, generally slow progression, and the need for ongoing care, and are often associated with irreversible changes that affect an individual’s functioning and daily life. Among the chronic diseases with the greatest impact on public health are Chronic Obstructive Pulmonary Disease (COPD) and Heart Failure (HF), owing to their high prevalence, morbidity, and mortality [3].

COPD is a heterogeneous lung disease characterized by chronic respiratory symptoms and progressive airflow limitation resulting from the interaction of genetic, environmental, and aging-related factors [4]. Beyond its physical manifestations, COPD has profound psychological, social, economic, and familial consequences for affected individuals [5]. In Portugal, it affects approximately 14% of the population aged 40 years and older [5]. HF, in turn, is a complex clinical syndrome resulting from structural and/or functional cardiac abnormalities that impair the heart’s ability to meet the body’s metabolic demands [6]. The often-unpredictable course of HF contributes to recurrent hospitalizations, progressive symptom deterioration, and reduced quality of life, affecting not only patients but also those who assume caregiving responsibilities [7]. In Portugal, its prevalence is estimated at 5.2%, with a significant increase expected due to population aging and the growing burden of comorbidities [8].

As both conditions progress to advanced stages, individuals face a range of physical, emotional, social, and existential challenges that can significantly compromise their well-being and quality of life [9]. In this context, Palliative Care (PC) plays a crucial role in addressing the complex needs associated with advanced chronic diseases [10]. The WHO defines PC as an approach aimed at improving the quality of life of people with PC needs and their families through the prevention and relief of suffering, achieved by means of early identification, assessment, and treatment of physical, psychological, social, and spiritual problems [11]. Consequently, the early integration of PC into disease trajectories has increasingly been recognized as an essential need, enabling a more individualized and person-centred response to care [12,13].

The philosophy of PC recognizes the individual as part of a broader relational system, placing particular emphasis on the role of family members and significant others [14]. In this context, the concept of family extends beyond biological or legal ties to encompass all individuals identified by the patient as significant within their life and illness journey [15]. The relationship between the patient and the family caregiver can be understood through the concept of the dyad, defined as a relational unit characterized by emotional bonds, mutual trust, and interdependence [16]. This perspective is particularly relevant in the context of advanced illness, as the experience of disease is not lived in isolation but rather shared and influenced by the interactions between both members of the dyad [17]. Therefore, understanding the dyad as a unit of care is essential for developing interventions that are more responsive to its emotional, communicational, and relational needs.

Among the many factors that influence the experience of advanced illness, hope has emerged as a central dimension of human experience in PC settings [18,19]. Far from representing merely an expectation of cure or life prolongation, hope is a dynamic and multidimensional life force that enables individuals to find meaning in their experience, cope with uncertainty, and identify possibilities for continuity in the face of adversity [20]. In the context of advanced illness, hope plays a significant role in adapting to physical, emotional, and existential suffering, contributing to the preservation of well-being and quality of life for both patients and family caregivers [21]. However, throughout the illness trajectory, hope may be continually challenged by symptom progression, loss of function, prognostic uncertainty, and changes in family dynamics. Consequently, the experience of hope becomes an ongoing process of reconstruction, through which the dyad seeks to reinterpret goals, redefine expectations, and find new meanings in their lived reality [21]. Healthcare professionals play a pivotal role in recognizing and supporting experiences of hope by fostering opportunities for dialogue and reflection that are sensitive to the individual and relational needs of each dyad.

Despite the growing scientific interest in hope within the context of advanced illness, the literature highlights a scarcity of qualitative studies exploring this phenomenon from the lived experiences of patients and their family caregivers. Furthermore, most studies have examined the perspectives of patients and caregivers separately, providing limited knowledge regarding hope as a shared experience within the dyad [22,23]. Given the relational nature of the advanced illness experience and the significance of hope in mitigating suffering, a deeper understanding of this phenomenon from a dyadic perspective is warranted.

Although Befecadu et al. [24] previously investigated hope among dyads living with advanced chronic illness, the present study differs in both sample and purpose. Whereas their longitudinal mixed-methods study primarily explored trajectories and correlates of hope over time, the current study adopts Amedeo Giorgi’s descriptive phenomenological methodology to gain an in-depth understanding of the lived meaning and essential structure of hope as experienced by patient–caregiver dyads. Therefore, the present study aimed to describe the lived experience of hope among dyads comprising individuals with advanced illness and their family caregivers, exploring the meanings they attribute to hope, the factors that strengthen or threaten it, and the ways in which it is experienced and sustained throughout the illness trajectory. Accordingly, the following research question was formulated: How do patient–caregiver dyads experience and sustain hope while living with advanced chronic illness? By exploring this experience, the study seeks to contribute to a deeper understanding of hope in the context of advanced illness, identifying elements relevant to clinical practice, professional healthcare education, and the development of interventions aimed at promoting the well-being and quality of life of dyads.

## 2. Materials and Methods

### 2.1. Study Design

This is a qualitative study, as it seeks to understand and describe the meanings individuals attribute to their lived experiences of a particular phenomenon [25]. The study adopts Giorgi’s descriptive phenomenological approach, which aims to understand and describe the essential structure of a phenomenon through participants lived experiences [26,27]. Grounded in Edmund Husserl’s phenomenology, this approach seeks to capture the meaning of experience as it is lived by the individual, while suspending, as far as possible, the researcher’s assumptions, prior knowledge, and interpretations through the adoption of the phenomenological attitude and phenomenological reduction [26,27]. This approach was considered appropriate for the aims of the study, as it seeks to explore the lived experience of hope among dyads composed of individuals with advanced illness and their family caregivers. Descriptive phenomenology enables an in-depth exploration of the meanings attributed to hope by participants and the identification of the essential structures that characterize this phenomenon in the context of advanced illness.

To ensure methodological rigor and quality in qualitative research, the study was conducted and reported in accordance with the Standards for Reporting Qualitative Research (SRQR) guidelines [28] (Supplementary Table S1).

### 2.2. Setting and Participants

The study was conducted in collaboration with the Pulmonology and Cardiology Departments of a hospital belonging to the Local Health Unit of the Leiria Region, Portugal. This public hospital is a major healthcare provider in central Portugal. Both departments regularly care for individuals with chronic respiratory and cardiovascular diseases, including patients with advanced COPD and HF, making them appropriate settings for identifying participants with direct experience of the phenomenon under investigation.

Given the phenomenological nature of the study and the need to obtain rich descriptions of the lived experience of hope, eligibility criteria were established to ensure the inclusion of participants with direct and meaningful experience of the phenomenon. Using purposive sampling, dyads composed of a patient and their primary family caregiver or significant other were recruited. A significant other was defined as a person with a relevant familial or emotional relationship with the patient and active involvement in caregiving [29].

Patients were eligible for inclusion if they met the following criteria: (1) diagnosis of advanced COPD (GOLD [Global Initiative for Chronic Obstructive Lung Disease] stage III with at least one hospitalization in the previous six months, or GOLD stage IV) [30], or diagnosis of advanced HF classified as New York Heart Association (NYHA) functional class III or IV, with at least one hospitalization due to disease exacerbation in the previous six months [31]; (2) aged 18 years or older; and (3) a score greater than 50% on the Palliative Performance Scale (PPS), ensuring adequate physical and cognitive capacity to participate in the interview.

Family caregivers were eligible if they: (1) were aged 18 years or older; and (2) were identified as the patient’s primary caregiver. Patients and caregivers were excluded if they had a mental health condition that impaired their ability to participate or if they were unable to communicate effectively. Additionally, patients presenting two or more symptoms with an intensity greater than 8 on the Edmonton Symptom Assessment Scale (ESAS) were excluded to avoid excessive physical or emotional burden associated with study participation.

With the aim of achieving maximum variation and ensuring the inclusion of diverse perspectives on the experience of hope, no restrictions were imposed regarding sex, age, sociocultural background, or time since diagnosis. This sampling strategy was intended to support a richer and more comprehensive understanding of the phenomenon within the context of advanced chronic illness. Data collection and phenomenological analysis were conducted concurrently, and recruitment decisions were continuously informed by emerging findings. Following regular discussions among the research team, recruitment ceased when interviews provided increasingly convergent descriptions of the meanings and essential dimensions of hope, and additional interviews no longer contributed substantively to the developing phenomenological structure. In line with Giorgi’s methodological perspective [27], emphasis was placed not on achieving thematic saturation but on attaining sufficient experiential depth, richness, and variation to support a rigorous description of the phenomenon’s essential structure. The final sample comprised 13 patient–caregiver dyads, including individuals living with advanced COPD or HF and their respective family caregivers.

### 2.3. Data Collection

Data collection took place between November 2025 and February 2026. Potential participants were initially approached by the head of each department, who provided information about the study’s purpose, procedures, and participation requirements. Individuals who expressed interest in participating were asked to provide their contact details and were given the written informed consent form. Following the receipt of signed informed consent, the principal investigator contacted participants by telephone to schedule the interview at a date and time convenient for them. Interviews were arranged according to participants’ availability and conducted in a location of their choice to promote comfort, privacy, and a sense of security.

Semi-structured interviews were conducted using an interview guide developed by the research team based on a review of the literature, with the aim of obtaining detailed and in-depth information regarding participants’ experiences and perspectives. The interview guide addressed the following topics: (i) meanings of hope in the experience of advanced illness; (ii) sources and everyday anchors of hope; (iii) challenges and threats to maintaining hope; and (iv) strategies used to cultivate and preserve hope in the face of adversity (Supplementary Table S2). Each interview began with the following open-ended question: “How do you describe your experience of hope while living with an advanced chronic illness?” Additional exploratory questions were used flexibly throughout the interviews according to emerging themes and the particular circumstances of each dyad (e.g., How did you feel? How did you deal with the situation? Can you give an example?). The interview guide was reviewed by two researchers with expertise in qualitative research (C.L. and A.Q.). This process was essential for identifying potential errors, inaccessible language, inconsistencies, redundant questions, and other aspects considered relevant to the evaluation of the instrument, thereby ensuring data collection aligned with the study objectives.

All interviews were conducted jointly with both members of the dyad by the principal investigator (L.F.C.), a cisgender woman and master’s student in PC, who had received specific training in qualitative interviewing techniques, including active listening, empathic understanding, management of silence, the use of open-ended questions, and validation of participants’ feelings. Joint interviews were selected because the study sought to explore hope as a relational phenomenon constructed within the patient–caregiver dyad. This approach enabled participants to jointly reflect on their experiences, elaborate on one another’s accounts, and reveal relational processes that might not emerge during individual interviews.

Throughout data collection, the interviewer adopted a stance of openness, respect, and sensitivity toward participants’ experiences, adapting her communication and interactions to the characteristics and sociocultural context of each dyad. Given the dyadic interview format, particular attention was paid to ensuring balanced participation. Both members were actively encouraged to share their perspectives, quieter participants were invited to contribute, and interruptions or interactions that limited participation were managed sensitively to ensure that neither voice dominated the discussion. Instances of agreement, disagreement, or differing viewpoints were explored respectfully and considered valuable opportunities to deepen understanding of the dyadic experience of hope. This flexible and responsive approach facilitated the expression of lived experiences and contributed to a richer understanding of the phenomenon under investigation [32].

When sensitive topics, such as disease progression, mortality, or emotional distress, emerged, participants were given time to reflect, decline questions, or temporarily pause the interview if desired. Particular attention was also paid to patients’ physical condition, including signs of fatigue, dyspnoea, or discomfort. When necessary, interviews were adapted through breaks, pacing adjustments, or early termination to safeguard participants’ well-being and comfort. No prior relationship or dependency existed between the researcher and the participants.

All interviews were conducted and audio-recorded in Portuguese. Sociodemographic information was collected to characterize the dyads, and field notes were used to document contextual observations, non-verbal interactions, interviewer reflections, and other elements considered relevant to data interpretation. Interviews were subsequently transcribed verbatim by the principal investigator, ensuring fidelity to participants’ accounts and preserving the nuances of their expressions to support rigorous phenomenological analysis. Each dyad was interviewed once, and transcripts were not returned to participants for comments or validation to avoid imposing additional burden on individuals living with advanced chronic illness and their family caregivers.

Quotations included in the manuscript were translated from Portuguese into English by bilingual members of the research team (L.F.C. and C.L.) and independently reviewed by the remaining authors to ensure semantic accuracy and preservation of experiential meaning. Particular attention was given to maintaining the phenomenological significance and contextual nuances of participants’ narratives rather than producing literal word-for-word translations.

### 2.4. Data Analysis

Data were analysed using Amedeo Giorgi’s descriptive phenomenological method [26], a rigorous approach grounded in the exploration of lived experience and the meanings individuals attribute to it [33,34]. The analysis followed six sequential steps: (1) collection and description of data through semi-structured interviews; (2) repeated reading of transcripts to gain a holistic understanding of participants’ accounts; (3) identification of meaning units; (4) transformation of these meaning units into psychologically sensitive and scientifically grounded expressions; (5) identification of the essential structure of the phenomenon; and (6) synthesis of the transformed meanings into a comprehensive description of the lived experience of hope. This approach enabled an in-depth exploration of the essence of hope among dyads composed of individuals living with advanced chronic illness and their family caregivers.

Although interviews were conducted jointly, meaning units were initially identified from each participant’s individual contributions within the shared interview transcript. These units were subsequently examined within the broader relational context of the dyadic interaction to identify shared meanings, complementary perspectives, jointly constructed accounts, and points of divergence. Particular attention was given to moments of agreement, disagreement, mutual elaboration, and relational positioning, as these interactions provided valuable insights into how hope was experienced, negotiated, and sustained within the dyad. Divergent perspectives were retained as meaningful dimensions of the phenomenon rather than treated as inconsistencies, thereby contributing to a richer understanding of the dyadic experience of hope and informing the development of the final phenomenological structure.

The primary analysis was conducted by L.F.C. and C.L., both trained in qualitative research and descriptive phenomenology. Meaning units, transformed meanings, and emerging constituent structures were discussed regularly during research team meetings to enhance analytical rigor and reflexive awareness. Differences in interpretation were resolved through collaborative discussion until consensus was reached, while maintaining close fidelity to participants’ descriptions and the principles of phenomenological reduction. Data organization, management, and analytical support were facilitated using WebQDA qualitative data analysis software.

### 2.5. Rigor and Reflexivity

To ensure methodological rigor and trustworthiness, the study was guided by the criteria of credibility, transferability, dependability, and confirmability [35]. Credibility was enhanced through prolonged engagement with the data, investigator triangulation, regular peer debriefing, reflexive discussions among the research team, and the use of representative participant quotations to support the findings. Transferability was promoted by providing a rich and detailed description of the study context, participants, and lived experiences, enabling readers to assess the applicability of the findings to other settings. Dependability was strengthened through the systematic and transparent application of Giorgi’s descriptive phenomenological method and through maintaining a clear record of analytical procedures and decisions. Confirmability was supported by ongoing reflexivity, collaborative analysis, and efforts to ensure that interpretations remained grounded in participants’ accounts rather than researchers’ assumptions.

Consistent with Husserlian descriptive phenomenology, researchers sought to adopt the phenomenological attitude through continuous reflexive practice and bracketing of pre-understandings. Prior to data collection and analysis, members of the research team identified, documented, and critically discussed their assumptions regarding hope, advanced chronic illness, caregiving, and PC. Reflexive notes were maintained throughout the study to increase awareness of potential influences arising from personal, clinical, and research experiences.

The research team comprised the principal investigator (L.F.C.), three researchers with clinical experience in the care of individuals with advanced chronic illness (E.G., S.P., and F.F.-R.), and two researchers with expertise in PC and qualitative research (C.L. and A.Q.). Investigator triangulation was achieved through the involvement of researchers with complementary clinical and methodological expertise in data interpretation. Emerging findings, meaning units, and phenomenological transformations were regularly reviewed during team meetings, fostering critical dialogue and challenging individual interpretations. In addition, periodic peer debriefing sessions were conducted to examine analytic decisions, explore alternative explanations, and ensure consistency with the data.

An audit trail was maintained throughout the study, documenting key methodological and analytical decisions, coding developments, reflexive observations, and discussions among team members. This process enhanced the transparency and auditability of the analysis. The rigor of the study was further strengthened through repeated engagement with the transcripts, collaborative examination of emerging meanings, and the presentation of representative quotations that illustrated the essential constituents of the phenomenon and allowed readers to evaluate the relationship between the data and the interpretations derived from them.

### 2.6. Ethical Considerations

Ethical approval for this study was granted by the Ethics Committee of the Local Health Unit of the Leiria Region (Approval No. 86/CEULSRL/2025 approved on 10 November 2025). The study was conducted in accordance with ethical principles governing research involving human participants and complied with the Declaration of Helsinki. Prior to data collection, all participants received detailed information regarding the study objectives, the voluntary nature of participation, their right to withdraw at any time without consequences for their healthcare, the measures adopted to ensure data protection, and the absence of any financial compensation. Confidentiality and anonymity were ensured using alphanumeric identifiers assigned to each dyad and individual participant (e.g., D1-PT, D1-CG), with access to audio recordings and transcripts restricted to the research team. Given the sensitive nature of the topics explored, including advanced illness and experiences of hope, interviews were conducted with particular attention to participants’ emotional well-being. A respectful, empathetic, and supportive environment was maintained throughout the data collection process to facilitate participants’ comfort and minimize potential distress.

## 3. Results

### 3.1. Description of Dyads

The sample comprised 26 participants, corresponding to 13 dyads consisting of individuals with advanced chronic disease and their respective family caregivers (Table 1). Of these, five dyads included a person diagnosed with COPD, and eight included a person diagnosed with advanced HF. The age of the individuals with chronic disease ranged from 48 to 82 years (mean ± SD: 73 ± 9 years), while the age of the family caregivers ranged from 41 to 80 years (mean ± SD: 66 ± 12 years). Regarding the relationship between dyad members, two types were identified: couple and father/daughter relationships. Time since diagnosis ranged from 3 months to 28 years.

### 3.2. Interview Findings

The findings were organized into four constituents, which were further divided into fifteen sub-constituents (Table 2). The first constituent, “Hope as a Horizon of Possibilities,” reflects a meaningful experience shared by the dyads, functioning as an essential resource for coping with the challenges associated with advanced illness. This hope is sustained by various sources, including family support, relationships with healthcare professionals, spiritual beliefs, and the perception of improvements or stability in health status, as described in the second constituent, “Everyday Anchors of Hope.” The third constituent, “Challenges to Sustaining Hope in Everyday Life,” highlights how hope is frequently threatened by disease progression, uncertainty about the future, suffering, and the emotional burden experienced by dyads. In response to these challenges, the fourth constituent, “Cultivating Hope in the Face of Adversity,” reflects the strategies used to maintain hope, including setting realistic goals, valuing everyday achievements, seeking social and emotional support, and focusing on the present moment. Taken together, the meanings, sources, threats, and strategies associated with hope constitute a dynamic experience that enables dyads to adapt to their circumstances and maintain a positive outlook in the face of adversity.

#### 3.2.1. Hope as a Horizon of Possibilities

Hope is a complex and multidimensional phenomenon, particularly relevant in the context of advanced chronic illness. Faced with disease progression, functional limitations, and uncertainty about the future, dyads rely on hope as an essential resource for coping with suffering and the challenges of everyday life. Four sub-constituents emerged from the analysis of the dyads’ narratives: (a) waiting for a transplant or cure; (b) having health and quality of life; (c) living one day at a time; and (d) spiritual peace and acceptance of destiny. These meanings reflect different ways of experiencing hope, ranging from the expectation of recovery to the search for meaning and adaptation in the face of illness.

(a) Waiting for a Transplant or Cure

In the context of advanced chronic illness, hope was often associated with the possibility of a cure, particularly through transplantation. More than a medical intervention, this possibility represented an opportunity to relieve suffering, regain autonomy, and rebuild life plans affected by the disease.

*“The hope is that, when the time comes for the transplant, everything will go well and the situation will improve compared to what it is now (…). We never thought twice when they told us that the solution would be a transplant (…). We hope that life will be different, without the machines (…).” (D7-PT)*

Despite uncertainty regarding clinical outcomes, the expectation of a cure or significant improvement in health status emerged as an important source of hope, fostering a more positive outlook on the future and enhancing the ability to cope with everyday adversities.

*“When we think about the possibility of a cure, we feel hopeful. It is an opportunity to regain some abilities and to do things that the illness has gradually taken away from us.” (D13-CG)*

(b) Having Health and Quality of Life

Hope was often expressed as the desire to preserve well-being and maintain disease stability, enabling participants to continue engaging in meaningful daily activities despite the limitations imposed by illness. In this context, having health did not necessarily mean being free from illness, but rather achieving a state of health that allowed them to live with comfort and dignity. Experiences such as performing simple daily tasks and spending time with family and friends were perceived as ways of maintaining quality of life.

*“In general, having hope means being able to have better health and a better quality of life.” (D2-PT)*

*“We believe that the greatest hope we can have is health. And when you have health, everything else becomes easier.” (D6-CG)*

*“For us, living well day by day is what matters most. And thinking a little about tomorrow, because we also have to think about it. But our hope is to live the coming years with health (…). Without health, we cannot do anything.” (D8-CG)*

(c) Living One Day at a Time

Participants frequently described hope as not primarily centred on the future but as the ability to live one day at a time. In the context of advanced chronic illness, the unpredictability of disease progression and uncertainty about the future led dyads to focus on the present, establish short-term goals, and value small daily achievements.

*“Hope? Well, it is about going to bed at night and hoping to wake up the next day and start a new day. There is nothing special. There are no improvements, much less a cure, so what matters is being well each day…” (D1-PT)*

*“As for the rest, whatever comes, comes. We have to accept it, whether good or bad, one day at a time, that is all. At this stage of life, we do not expect much anymore.” (D6-PT)*

*“It is one day at a time; there is not much more we can do.” (D9-PT)*

*“In our case, it is really about living one day at a time and staying strong.” (D11-CG)*

This way of experiencing hope reflects an adaptation to the illness process, enabling dyads to avoid excessive concern about the future and focus instead on what they are able to control in the present. Overall, this shared definition of hope highlights the importance of living in the present moment as a strategy for coping with the adversities associated with illness.

(d) Spiritual Peace and Acceptance of Destiny

The narratives also revealed that hope tended to assume a more existential character, associated with accepting reality, finding meaning in the experience of illness, and trusting in a purpose that transcends immediate suffering. This form of hope is marked by the integration of illness into one’s life story, allowing dyads to face the present and the future with greater serenity and a sense of continuity.

*“Deep down, we have to keep hope alive (…). And then there is another kind of hope, deeper, more inward. It is difficult to explain… it is like a feeling that everything does not end here, that life has a greater meaning, even in the face of illness (…).” (D1-CG2)*

*“Before, hope was very much linked to the future; today it is not quite like that. Now it is more connected to the way we look at life (…)” (D13-PT); “It is about accepting life as it is, with the good things and the bad things, because everything is part of it (…)” (D13-CG); “Because, if we are realistic, nothing will ever be the way it was before” (D13-PT); “And hope is exactly that: continuing to live with what we have and finding peace.” (D13-CG)*

This exchange illustrates how meanings of hope were jointly negotiated within the interview, with both members complementing one another’s interpretations and constructing a shared understanding of hope as acceptance and adaptation.

#### 3.2.2. Everyday Anchors of Hope

Analysis of the narratives revealed that hope is sustained by several factors that contribute to its preservation throughout the illness trajectory. Faced with the challenges associated with living with an advanced chronic illness, dyads draw upon different internal and external resources that enable them to find strength, meaning, and motivation to cope with adversity and the suffering inherent to their condition. Four main anchors of hope emerged from the participants’ accounts: (a) resilience and inner strength; (b) social interactions and friendships; (c) family, legacy, and a sense of continuity; and (d) faith and religious/spiritual beliefs. These sources constitute fundamental pillars in maintaining hope and providing support and meaning in the face of uncertainty and the limitations imposed by illness.

(a) Resilience and Inner Strength

Through resilience and inner strength, dyads seek to adapt to the changes and limitations imposed by illness, to cope with suffering, and simultaneously find meaning and purpose in life.

*“Nothing makes us lose hope.” (D3-CG)*

*“We never completely lose hope. People usually say that hope is the last thing to die. The day may be grey and full of clouds, but it is still worth continuing to move forward.” (D4-PT)*

*“Hope is never lost, we keep moving forward, whatever happens. That is how we think.” (D7-CG)*

Some dyads also associated the preservation of hope with previous life experiences and adversities they had already overcome. Difficult moments experienced throughout their lives appeared to have contributed to the development of adaptive skills, strengthening their resilience. Even when confronted with the challenges associated with disease progression, they sought to maintain an optimistic attitude and sustain hope.

*“Despite the difficulties and challenges we face every day, we try to find within ourselves the ability to move forward, support one another, and never give up.” (D6-CG)*

*“We have already been through many adversities and have always kept hope alive.” (D8-PT)*

(b) Social Interactions and Friendships

Disease progression may lead to social isolation, because the focus on care and illness-related limitations tends to reduce opportunities for social interaction. In this context, maintaining a social network and supportive relationships becomes particularly important, contributing to reduced suffering, a stronger sense of belonging, and feelings of being supported and valued. Social interactions allow dyads to remain connected to their social environment and preserve their identity.

*“When I go to the café and spend time with my friends… that brings me joy. It is that little moment I have for myself.” (D3-PT)*

Some participants reported that social gatherings constitute a source of hope because they create opportunities to share experiences, promote emotional well-being, and help divert attention from illness-related concerns.

*“We have a neighbour with whom we often have long conversations. She shares her worries, and we share ours (…)” (D4-CG); “And in the end, we feel some relief” (D4-PT); “We know that nobody can cure us, but talking helps” (D4-CG); “It lightens the soul (…) and gives us hope” (D4-PT)*

*“We need to talk and share things with each other and with those around us, especially family and close friends (…). From time to time, we have lunch together when the weather is nice. We need to spend time together.” (D13-CG)*

(c) Family, Legacy, and a Sense of Continuity

Family relationships and emotional bonds emerged as some of the main sources of hope for the dyads. The support of a spouse, the presence of family members, and particularly relationships with grandchildren were identified as important sources of strength, motivation, and meaning in life. The possibility of watching grandchildren grow, maintaining family connections, and feeling useful to loved ones helped sustain hope and cope with the difficulties associated with illness.

*“We have always been each other’s support, and hope to continue being so (…)” (D1-PT); “After 58 years of walking through life together, that has never changed” (D1-CG); “We know that, if we need anything, we always have each other” (D1-PT); “That presence is always there.” (D1-CG)*

*“My hope is in our granddaughter. I want her to achieve everything she dreams of. I hope she will be successful in life because she has been a great source of strength for us.” (D3-PT)*

*“That little girl truly became our hope (…). Our granddaughter brought so much life into our lives, so much indeed. At the beginning, everything was very difficult.” (D4-CG)*

*“When I feel down, we think about our family, our grandchildren, and the small things I can still do. That is what gives me strength (…). Sometimes, if it were not for them, everything would be much harder.” (D11-PT)*

In addition, some dyads associated hope with building and preserving a legacy by passing on values, memories, and experiences to future generations. This concern with leaving a positive mark on the lives of others enabled them to find meaning in their life journey and preserve their identity beyond illness.

*“My father passed away at almost 93 years old (the patient’s father), but I still keep the local newspaper subscription in his name. It gives me pleasure to say that this is my father’s name. Many people get rid of such things immediately… I like to preserve his memory.” (D6-PT)*

*“Everyone experiences hope differently, but for us, what matters is everything we have lived, the people we have met, and the values we have passed on, especially to our children (…). Life is built over time, and for us it is very important to give it meaning because, otherwise, what are we doing here? Hope is exactly that: leaving something of ourselves to others, especially our family and the people closest to us.” (D13-PT)*

Thus, hope is not limited to the expectation of clinical improvement. Rather, it is strongly linked to emotional bonds, a sense of family belonging, and the desire to leave a legacy, all of which promote emotional well-being and reinforce the dyads’ sense of purpose.

(d) Faith and Religious/Spiritual Beliefs

Faith and religious/spiritual beliefs also emerged as important sources of hope, particularly in the face of uncertainty about the future. For many dyads, belief in God, the divine, or a higher power constituted a fundamental resource for coping with the challenges associated with illness and for providing comfort, security, inner strength, and meaning in life. Furthermore, some dyads expressed the conviction that their suffering had a purpose or meaning, believing that life’s journey is guided by a higher will. This perspective appeared to facilitate acceptance of their illness and contribute to sustaining hope.

*“As long as God gives us strength and courage, we will keep fighting.” (D10-PT)*

*“We feel that this is not the end, that life has a greater meaning, even with illness. That gives us great peace of mind.” (D12-CG)*

In addition to beliefs, religious and spiritual practices were also identified as important sources of hope. Activities such as daily prayer, reciting the rosary, and participating in religious services were described as strategies that promote emotional comfort and strengthen faith. These practices provide opportunities for reflection and connection with the transcendent, allowing individuals to express fears, concerns, and wishes, thereby alleviating suffering and reinforcing feelings of hope and trust in the face of adversity.

*“Going to Mass and praying are moments when we connect with God, and that relieves our suffering.” (D6-CG)*

#### 3.2.3. Challenges to Sustaining Hope in Everyday Life

Throughout the illness trajectory, several factors may challenge the maintenance of hope, generating feelings of frustration, fear, insecurity, discouragement, and helplessness. The main challenges identified by the dyads comprised the following sub-constituents: (a) progressive disease deterioration; (b) caregiver burden; (c) loss of loved ones; and (d) loneliness and social abandonment.

(a) Progressive Disease Deterioration

Progressive disease deterioration was identified by the dyads as one of the main threats to hope. The course of the illness, marked by increasing symptoms, physical limitations, and dependence on others, generates suffering and fosters feelings of frustration, helplessness, and discouragement.

*“It is a disease that, day after day, brings more difficulties and reduces my hope for the future.” (D1-PT)*

*“Everything happened so quickly, and it was difficult to keep up with all the changes.” (D10-CG)*

*“It was difficult to accept the illness and the loss of independence, especially when we became more dependent on each other.” (D11-PT)*

(b) Caregiver Burden

Caregiver burden emerged as an important challenge to the hope of the dyads. As the disease progresses and the patient’s needs increase, family caregivers take on increasingly demanding responsibilities related to constant monitoring, care management, and attendance at medical appointments and examinations. This reality often results in physical exhaustion, emotional strain, and limitations in personal life, compromising caregivers’ well-being and their ability to maintain a hopeful outlook.

*“Sometimes the strength to carry on is lacking because fatigue and burden become overwhelming. It has not been easy.” (D2-PT)*

*“The demands of daily life are increasing all the time and, sometimes, you no longer have the capacity to cope with everything.” (D4-CG)*

In addition to the demands of caregiving, caregivers reported feelings of worry, fear, and helplessness regarding disease progression and the patient’s gradual loss of autonomy. The fear of not being able to adequately meet the patient’s needs, together with accumulated exhaustion, contributes to emotional distress and weakens hope.

*“There are times when I break down, I will not lie. Especially when I see her getting worse… it hurts a lot. You know that feeling of wanting to do something and not being able to? That’s it, it really hurts. And there is also the accumulated fatigue, sometimes you just feel completely drained.” (D12-CG)*

*“For me, this has been a period of great learning. Because, besides caring for him, I accompany him every day and everywhere. My fear is that I may not be capable of helping him.” (D13-CG)*

(c) Loss of Loved Ones

The loss of loved ones was identified as a threat to hope, as it constitutes a profound experience that intensifies emotional suffering and evokes feelings of sadness, insecurity, and vulnerability. The loss of significant individuals, especially close family members who played a supportive role, confronts dyads with the finitude of life and may weaken the resources that sustain hope.

*“The loss of a child remains a deep pain that still influences the way we live and cope with suffering today.” (D2-CG)*

*“Losing a child is one of the most difficult experiences we can face, and it leaves a mark that remains over time.” (D5-PT)*

Nevertheless, some dyads demonstrated the ability to find meaning in these experiences of loss by valuing their life journey and redefining personal goals, which contributed to the reconstruction of hope.

*“Life is built over time and, for us, it is very important to give it meaning in order to cope with the losses we have experienced throughout life (…).” (D13-CG)*

(d) Loneliness and Social Abandonment

Loneliness and social abandonment were identified by the dyads as factors that threaten the maintenance of hope. The limitations imposed by advanced illness, combined with reduced social interactions and the absence of a close support network, foster isolation and feelings of abandonment. This reality generates feelings of sadness, devaluation, and vulnerability, making it more difficult to cope positively with the challenges of illness.

*“I took care of both my parents and had to close my business… it was difficult (…). Even harder is having siblings who live nearby and are not capable of visiting their parents (…). That really shocks me.” (D5-CG)*

*“I feel trapped, I cannot go anywhere (…). Sometimes I am overwhelmed by a profound sense of loneliness (…) He feels that way too…” (D10-CG)*

Furthermore, the absence of family support, lack of assistance with caregiving responsibilities, and limited opportunities to share concerns and emotions intensified feelings of abandonment. In this context, the lack of emotional and social support weakened hope by depriving dyads of important sources of security, comfort, and encouragement.

*“It is just the two of us. We do not have relatives who support us. We feel that there is no one we can turn to or confide in (…). We have family nearby, but it is as if we did not (…). Deep down, we feel alone and abandoned.” (D2-PT)*

#### 3.2.4. Cultivating Hope in the Face of Adversity

Throughout the illness trajectory, dyads developed a range of strategies that contributed to maintaining hope, enabling them to adapt to adversity and continue viewing life in a positive way. Three main sub-constituents emerged from the participants’ narratives: (a) living in the present and making the most of life; (b) dialogue and open communication within the dyad; and (c) accepting support from others.

(a) Living in the Present and Making the Most of Life

Living in the present moment and making the most of life emerged as an important strategy for sustaining hope. Many dyads chose to focus on the present, on available opportunities, and on appreciating positive moments, rather than becoming overwhelmed by concerns about the future and the potential consequences of disease progression. In this context, hope became associated with the ability to value small daily achievements, engage in meaningful activities, and maintain close relationships with significant others, all of which were considered essential for preserving comfort and well-being throughout the illness trajectory.

*“What I would recommend is enjoying life while there is still time, because it comes to an end. Life goes by (…). Sometimes it is short. And I think that is what I would recommend, enjoying life. I think it is the best thing we can do. While there is still time.” (D9-PT)*

*“To value the present more, because dealing with illness is difficult, but it also helps us grow. We cannot spend all our time thinking about it; otherwise, we forget to live. Life is lived both with and without illness.” (D13-PT)*

(b) Dialogue and Open Communication Within the Dyad

Open communication was identified as an important promoter of hope. Sharing feelings, concerns, and expectations strengthened trust, mutual support, and emotional bonds, facilitating the management of everyday challenges. Moreover, feeling heard and understood by the other person contributed to the construction of a shared vision of the future and to the maintenance of both hope and well-being within the dyad.

*“We cannot lose hope, because then everything becomes worse (…), but talking openly makes everything easier.” (D11-PT)*

*“Although it is difficult, we have managed to live with the illness. Life is not the same as before, but it has helped bring us closer together and strengthen our bond.” (D12-CG)*

*“One of the things that changed because of this illness was that I started sharing what I feel and think, especially with my wife. I did not talk much before and kept many things to myself, now I never stop talking.” (D13-PT)*

(c) Accepting Support from Others

Accepting support from others emerged as an important strategy for fostering hope among the dyads. Recognizing the need for support and being willing to accept help from family members, friends, neighbours, or healthcare professionals contributed to reducing caregiver burden, strengthening feelings of security, and minimizing experiences of loneliness and social abandonment.

*“I think we would have to go back to the beginning and do things the way we did (…). If there is no better solution… it depends on the situation, but when someone is not managing to recover, I think they need to ask for help. At least in the hospital, I know they have support services.” (D9-CG)*

*“Seeking help, not only from family but also from doctors and nurses, because they know what we should do during the most difficult moments.” (D11-CG)*

In addition, support from a broader social network enabled caregivers to have more time for self-care and meaningful activities, thereby promoting well-being, quality of life, and the preservation of hope.

*“We have to accept help from others. Even when it seems small, it can make a huge difference in our daily lives, bringing us more peace of mind, well-being, and hope to keep going.” (D12-CG)*

## 4. Discussion

This study’s findings highlight hope as a dynamic, relational, and deeply embedded experience within the context of advanced chronic illness. Rather than constituting a solely individual resource, hope emerged as a phenomenon constructed within the dyad’s intersubjective space, shaped by the ongoing interaction between the patient and the family caregiver. Exploring the meanings attributed to hope, its sources, threats, and preservation strategies revealed its importance as a key resource for adaptation, well-being, and quality of life. Hope, understood as a “horizon of possibilities,” was experienced as a flexible and evolving phenomenon that adapted to the changing realities of the illness. This interpretation aligns with a previous study [24], which describes hope as a constantly evolving phenomenon that changes alongside disease progression and the shifting needs of dyads. Within this process, hope becomes less exclusively associated with the possibility of cure and acquires meanings that are better adapted to the realities of living with advanced illness. For some dyads, hope was expressed through expectations of transplantation or clinical improvement, reflecting the desire to regain autonomy, resume meaningful activities, and achieve a greater sense of normality in everyday life. As highlighted by Befecadu et al. [24], such expectations represent not only the pursuit of treatment but also aspirations for an improved quality of life. For other dyads, hope was centred on maintaining health and quality of life, expressed through symptom control, preservation of independence, and the ability to continue engaging in meaningful activities, thereby emerging as an essential condition for living with dignity, comfort, and well-being [36].

This reconfiguration of hope was also reflected in the notion of living one day at a time, a meaning likewise identified by Befecadu et al. [24], which emphasizes a focus on the present and on short-term goals and expectations. From this perspective, hope is nurtured by small daily achievements, meaningful moments, and the ability to meet immediate needs, helping dyads manage uncertainty and reduce excessive concern about the future [24,36]. Simultaneously, a spiritual and existential dimension of hope emerged, expressed through the pursuit of inner peace and the acceptance of disease progression and human finitude. However, such acceptance was not experienced as resignation or abandonment of hope but rather as an adaptive process through which meaning could be attributed to illness and its associated suffering. These findings support existing evidence recognizing spirituality as a central dimension of hope in advanced illness, contributing to spiritual well-being, the alleviation of existential suffering, and the strengthening of serenity and inner peace [37,38,39].

Regarding the second constituent, Everyday Anchors of Hope, resilience and inner strength emerged as fundamental sources of hope. Participants described hope as an enduring inner resource that enabled them to confront suffering, navigate uncertainty, and adapt to the challenges imposed by advanced illness while preserving a sense of meaning and purpose [36]. This finding is consistent with previous dyadic research, which highlights hope as a sustaining force that supports resilience and coping throughout the illness trajectory [24]. Within the dyadic context, resilience appeared to underpin the ongoing reconstruction and maintenance of hope in the face of changing circumstances and adversity [21,36].

Social interactions and friendships, together with affective bonds and legacy, also emerged as important sources of hope. Indeed, the bond established between members of the dyad, interpersonal relationships, and interactions with family and friends were found to be essential for maintaining and strengthening hope, with family occupying a central role in the construction of goals and the search for meaning [21,36]. Sharing experiences, receiving emotional support, and communicating with others facilitate adaptation to illness and contribute to preserving hope, while simultaneously helping patients feel valued and appreciated by those around them [18].

The desire to witness grandchildren growing up and concerns about leaving a positive legacy for future generations emerged as important promoters of hope. This perspective is consistent with the conceptualization of legacy proposed by Timóteo et al. [40], which emphasizes the importance of leaving something of value that transcends death and contributes to future generations as a means of finding meaning at the end of life. Complementing this perspective, the search for meaning associated with legacy was accompanied by a spiritual dimension of hope that functioned as an important coping resource. Faith and religious or spiritual beliefs were perceived as sources of hope because they provided comfort and enabled participants to find a purpose for continuing to live [38,39]. This finding is consistent with the previously identified meaning of hope as “spiritual peace and acceptance of destiny,” reinforcing the recognition of spirituality as an essential dimension of hope in advanced illness [38]. Similarly, Laranjeira et al. [18] found that hope is frequently associated with faith and belief in a higher power, functioning as an important resource for maintaining hope and alleviating existential suffering throughout the illness trajectory.

Despite the many factors promoting hope, the findings also revealed challenges that interfere with its maintenance throughout the disease trajectory. Progressive disease deterioration was one of the most frequently reported threats to hope. The diagnosis of an advanced illness, gradual loss of autonomy, and social isolation contribute to reduced expectations among both patients and caregivers who closely witness disease progression [41]. As symptoms intensify and the illness advances, hope may become increasingly vulnerable [18]. Nevertheless, this process does not necessarily imply the disappearance of hope. Rather, hope tends to be reformulated and redirected toward more realistic and attainable goals, such as symptom control, comfort, and the appreciation of the quality of time lived [42].

Caregiver burden also emerged as a significant challenge to hope. The physical, emotional, and social demands associated with caregiving substantially influence how hope is experienced. Dyads reported feelings of fatigue, worry, helplessness, and increased responsibility, which often made it more difficult to sustain a hopeful outlook. These findings are supported by Duggleby et al. [43], who identified an association between lower levels of hope and higher levels of psychological distress, anxiety, guilt, or anticipatory grief. Conversely, caregivers equipped with effective adaptive strategies, greater caregiving preparedness, and stronger personal management resources tended to report higher levels of hope [43].

The loss of significant others emerged as another factor that weakened hope. Recurrent losses, proximity to death, and experiences of grief represent considerable challenges to maintaining hope in the context of advanced illness. When confronted with the death of family members or close friends, dyads often perceived these events as reminders of their own vulnerability and mortality, contributing to increased emotional suffering. Qualitative studies suggest that such experiences may temporarily undermine hope and require a reformulation of its meaning, together with the mobilization of supportive resources to cope with illness and awareness of finitude [24,42].

Additionally, loneliness and social abandonment emerged as important threats to the exercise of hope. As illness progressed, dyads described reductions in social interactions and increasing feelings of isolation, frequently associated with the absence of people available to provide assistance or share concerns and difficulties. This lack of support compromises the ability to maintain a positive perspective. Indeed, the literature identifies social support as one of the main foundations of hope, while its absence contributes to greater psychological distress, hinders adaptation to illness, and weakens individuals’ capacity to view the future positively [42,43].

Despite the adversities threatening hope throughout the disease trajectory, dyads demonstrated the ability to mobilize personal and relational strategies that support its preservation. In response to uncertainty, loss, and the limitations imposed by illness progression, participants emphasized the importance of living in the present and making the most of life while possible. This strategy closely resembles the previously identified meaning of living one day at a time. According to Yousofvand et al. [44], individuals with advanced chronic illness and their caregivers tend to preserve hope by focusing on the present, valuing small daily achievements, and avoiding excessive concerns about an uncertain future. Maintaining hope therefore involves the ability to attribute meaning to the present moment, value interpersonal relationships, recognize personal strengths, and establish realistic short-term goals [36,45], thereby preserving a sense of normality and continuing to find value and purpose in life despite illness [20,44].

Beyond individual resources, hope was strongly influenced by the quality of interpersonal relationships. In particular, dialogue and open communication between members of the dyad emerged as fundamental strategies for coping with illness-related difficulties. Befecadu et al. [24] suggest that hope is experienced relationally and is continuously shaped through interactions between the patient and the family caregiver. Within this context, emotional bonds, mutual support, and the open sharing of concerns, feelings, and expectations contribute to strengthening hope for both members of the dyad, reinforcing the importance of interventions that address the patient and caregiver simultaneously [24].

Finally, accepting support from family members, friends, and healthcare professionals emerged as another important strategy for fostering hope. Given that caregiver burden, loneliness, and social abandonment were identified as factors that undermine hope, the availability of a supportive network appears to function as a protective resource [43,46]. Family, social, and professional support reduces the physical and emotional burden associated with caregiving, promotes feelings of security, and reinforces the perception that the dyad is not facing illness alone. Consequently, feeling supported contributes to maintaining hope while mitigating the loneliness and vulnerability often associated with advanced illness [18].

### 4.1. Strengths and Study Limitations

This study contributes to a deeper understanding of hope as a relational phenomenon experienced within patient–caregiver dyads living with advanced chronic illness. A key strength lies in its qualitative design, which enabled an in-depth exploration of the meanings attributed to hope, as well as its perceived sources, challenges, and sustaining strategies. The use of Amedeo Giorgi’s descriptive phenomenological methodology provided a rigorous framework for capturing the complexity and multidimensional nature of hope as it is lived and interpreted by participants. Furthermore, by examining the shared experiences of patients and family caregivers, the study advances knowledge regarding the intersubjective and relational dimensions of hope, reinforcing its relevance as an important resource for adapting to illness-related suffering and uncertainty.

Several limitations should nevertheless be acknowledged. The findings reflect the experiences of a relatively small group of dyads recruited within a specific sociocultural and healthcare context in Portugal, which may limit their transferability to other settings and populations. Participation may also have been influenced by self-selection bias, as individuals willing to discuss their experiences of illness and hope may differ from those experiencing greater distress or disengagement. Moreover, the subjective nature of hope, together with the influence of cultural, spiritual, familial, and biographical factors, requires that the findings be interpreted within the context in which they were generated.

Although the sample intentionally included participants with diverse illness histories and disease trajectories to maximize experiential variation, time since diagnosis may not accurately reflect the duration or severity of advanced illness. Consequently, participants may have differed substantially in their experience of living with advanced disease. Future research would benefit from incorporating more detailed indicators of disease trajectory, functional status, symptom burden, and specialist PC involvement. Additionally, contextual variables that may shape experiences of hope, such as duration of caregiving, cohabitation status, employment status, religiosity, and previous engagement with PC services, were not systematically collected. The absence of these data may have limited a fuller understanding of the personal, relational, and contextual factors influencing hope within the dyad. Future studies should consider including these characteristics to further contextualize the experience of hope in advanced chronic illness.

The eligibility criteria, including the requirement for a Palliative Performance Scale (PPS) score above 50% and the exclusion of patients with high symptom burden, were necessary to ensure participants’ ability to engage in an in-depth phenomenological interview while minimizing potential burden. However, these criteria may have reduced the representation of individuals experiencing more severe functional deterioration or distress. As a result, the findings may reflect relatively more adaptive experiences of hope than those of individuals living with greater physical vulnerability and symptom-related suffering.

Transferability may also be constrained by the composition of the sample, which consisted predominantly of couple relationships, with relatively limited representation of other caregiving arrangements. Experiences of hope within sibling dyads, adult child–parent caregiving relationships, or non-family caregiving networks may differ in important ways and warrant further investigation.

In addition, although joint interviews facilitated the exploration of hope as a relational and co-constructed phenomenon, the presence of both members of the dyad may have limited the expression of highly personal, sensitive, or potentially conflicting experiences and viewpoints. Similarly, transcripts were not returned to participants for member checking in order to avoid imposing additional burden on individuals living with advanced illness and their caregivers. While this decision was ethically justified, it may have reduced opportunities for participant validation of interpretations. To enhance the credibility and trustworthiness of the findings, alternative strategies were employed, including reflexive analysis, regular peer debriefing, investigator triangulation, and ongoing discussions among the research team throughout the analytical process.

Finally, because data were collected at a single point in time, the study could not directly capture how hope changes over the course of illness progression. Consequently, descriptions of changes in hope are based on participants’ retrospective narratives rather than on longitudinal observation. Future longitudinal phenomenological studies may provide further insight into the ways hope is maintained, reinterpreted, and negotiated across different stages of advanced chronic illness.

### 4.2. Implications for Practice

The findings of this study suggest that hope remains an important aspect of the experience of advanced chronic illness, although participants described its meanings as being reshaped in response to changing circumstances. This dynamic and multidimensional understanding of hope may have implications for clinical practice, particularly in PC and in the care of individuals with advanced chronic illness and their family caregivers. The findings highlight the potential value of integrating PC principles earlier in the illness trajectory, given the complexity of the physical, psychological, social, and spiritual challenges faced by both patients and caregivers. Owing to their expertise in addressing expectations, concerns, goals, and priorities, PC professionals may be particularly well positioned to support conversations and interventions that foster hope.

Understanding hope as a dynamic, relational, and context-dependent phenomenon also draws attention to its relevance within care planning. The findings suggest that hope is experienced not only individually but also within the relational context of the dyad, shaped by the interactions, experiences, and challenges encountered by both members. As such, a dyad-centred approach to care may help healthcare professionals better recognize the reciprocal influences between patients and caregivers and their potential impact on the ways hope is understood and maintained.

Furthermore, the findings suggest that healthcare professionals may benefit from considering not only the meanings attributed to hope but also its sources, perceived threats, and the strategies used to sustain it. Such understanding may inform individualized and responsive care that supports personal, relational, and spiritual resources while facilitating adaptation to the challenges associated with advanced illness. Creating opportunities for patients and caregivers to express their concerns, expectations, and life goals may also support the development of realistic and meaningful sources of hope. In addition, raising awareness of the role of hope in the illness experience and strengthening competencies related to communication, emotional support, and the assessment of social and spiritual needs may enhance person- and family-centred care [47,48]. Within this context, fostering hope may represent an important component of holistic palliative care practice.

Future research would benefit from longitudinal designs to explore how experiences and meanings of hope are reported across different stages of advanced illness and to examine the factors that may influence their reformulation over time. Further studies involving dyads with more diverse sociodemographic and clinical characteristics and from different healthcare settings may also broaden understanding of the contextual factors shaping hope. Finally, additional research is needed to develop and evaluate interventions aimed at supporting hope, particularly psychosocial, spiritual, and dyad-centred approaches. Such work may contribute to a stronger evidence base for hope-focused care and inform strategies to support adaptation, well-being, and quality of life among people living with advanced illness and their family caregivers.

## 5. Conclusions

This descriptive phenomenological study contributed to a deeper understanding of the lived experience of hope among dyads composed of individuals with advanced chronic illness and their family caregivers, highlighting the importance of addressing hope from a relational perspective. The findings revealed that hope is a dynamic, multidimensional, and continuously evolving phenomenon whose meanings are reshaped in response to disease progression and the changing circumstances of the dyads. Rather than representing solely an expectation of cure, hope emerged as an essential adaptive resource that enables individuals to find meaning in the illness experience, cope with uncertainty, confront losses, and value what remains meaningful throughout the disease trajectory. Within this sample, hope appeared to be closely linked to the quality of interpersonal relationships, emotional bonds, spirituality, personal beliefs and values, and the support provided by family members, friends, and healthcare professionals. At the same time, hope was shown to be vulnerable to challenges such as disease progression, caregiver burden, significant losses, and social isolation, requiring ongoing reformulation of its meanings and goals. Nevertheless, dyads demonstrated the ability to mobilize personal, relational, and spiritual resources that enabled them to preserve hope, even in situations of considerable vulnerability. Overall, the findings suggest that attention to hope may be an important component of PC for patients with advanced chronic illness and their family caregivers. Promoting hope requires acknowledging the uniqueness of each dyad, supporting its internal and relational resources, and creating conditions that enable individuals to continue finding meaning, purpose, and value in life despite disease progression and the awareness of finitude. More than an expectation about the future, hope was described as a process of adaptation and meaning-making through which participants sought to preserve dignity, connectedness, and a sense of purpose despite illness progression.

## Figures and Tables

**Table 1:** Participant Characteristics.

Dyad	Dyad Member	Gender	Age	Type of Relationship	Diagnosis	Time Since Diagnosis
1	Patient	Male	80	Couple	COPD	9 years
	Caregiver	Female	73			
2	Patient	Male	73	Couple	COPD	10 years
	Caregiver	Female	67			
3	Patient	Male	71	Couple	COPD	6 years
	Caregiver	Female	66			
4	Patient	Female	70	Couple	COPD	12 years
	Caregiver	Male	75			
5	Patient	Male	80	Father/Daughter	HF	1 year
	Caregiver	Female	52			
6	Patient	Female	80	Couple	HF	11 years
	Caregiver	Male	76			
7	Patient	Female	48	Couple	COPD	5 years
	Caregiver	Male	65			
8	Patient	Male	80	Couple	HF	28 years
	Caregiver	Female	41			
9	Patient	Male	74	Father/Daughter	HF	3 months
	Caregiver	Female	45			
10	Patient	Male	82	Couple	HF	4 months
	Caregiver	Female	80			
11	Patient	Male	82	Couple	HF	1 year
	Caregiver	Female	79			
12	Patient	Female	69	Couple	HF	8 months
	Caregiver	Male	70			
13	Patient	Male	65	Couple	HF	2 years
	Caregiver	Female	66			

Note: COPD: Chronic Obstructive Pulmonary Disease; HF: Heart Failure.

**Table 2:** An overview of the constituents and sub-constituents of lived experience of Hope.

Constituents	Sub-Constituents
Hope as a Horizon of Possibilities	Waiting for a transplant or cure
	Having health and quality of life
	Living one day at a time
	Spiritual peace and acceptance of destiny
Everyday Anchors of Hope	Resilience and inner strength
	Social interactions and friendships
	Family, legacy, and a sense of continuity
	Faith and religious/spiritual beliefs
Challenges to Sustaining Hope in Everyday Life	Progressive disease deterioration
	Caregiver burden
	Loss of loved ones
	Loneliness and social abandonment
Cultivating Hope in the Face of Adversity	Living in the present and making the most of life
	Dialogue and open communication within the dyad
	Acceptance of support from others

## Data Availability

The data that support the findings of this study are available from the corresponding author upon reasonable request. This article is based on the first author’s Master’s dissertation in Palliative Care at the School of Health Sciences—University of Leiria and Oeste.

## Supplementary Materials

The following supporting information can be downloaded at https://www.mdpi.com/article/10.3390/healthcare14182985/s1, Table S1: Standards for Reporting Qualitative Research (SRQR) checklist; Table S2: Semi-Structured Interview Guide.

## Abbreviations

The following abbreviations are used in this manuscript:

COPD	Chronic Obstructive Pulmonary Disease
GOLD	Global Initiative for Chronic Obstructive Lung Disease
ESAS	Edmonton Symptom Assessment Scale
HF	Heart Failure
NYHA	New York Heart Association
PC	Palliative Care
PPS	Palliative Performance Scale
