# Supplementary material for: Exploring Hope in Patient–Caregiver Dyads Living with Advanced Chronic Disease in Portugal: A Phenomenological Study
Source: Healthcare (Basel). 2026 Sep 12;14(18):2985. doi: 10.3390/healthcare14182985 (PMC13606639; doi:10.3390/healthcare14182985)
Supplement: Supplementary file 1 [file healthcare-14-02985-s001.zip › Supplementary Table S2_Interview_Guide.pdf]

Supplementary Table S2: Semi-Structured Interview Guide.

**Opening question**	1. To begin, could you describe what hope means to you while living with (or caring for someone with) an advanced chronic illness?
**Meanings of Hope in the Experience of Advanced Illness**	2. How do you experience hope in your daily life?<br>3. What are you hoping for at this stage of the illness?<br>4. Has the progression of the illness changed the meaning of hope for you? If so, how?
**Sources and everyday anchors of hope**	5. What helps you maintain hope during difficult times?<br>6. Who or what gives you strength, encouragement, or motivation to continue?<br>7. Are there particular people, relationships, beliefs, or activities that sustain your hope?<br>8. How do you influence one another's hope as a patient-caregiver dyad?<br>• In what ways do you support each other?<br>• Are there moments when one person's hope helps the other?
**Challenges and threats to maintaining hope**	9. What situations or experiences make it difficult to maintain hope? Can you give an example?<br>10. Have there been moments when your hope felt weakened or challenged?
**Strategies used to cultivate and preserve hope in the face of adversity**	11. What do you do to maintain hope when facing difficult situations?<br>12. Have you developed any personal or family strategies that help you cope with adversity?
**Closing Question**	13. Looking back on your journey, what has hope allowed you to do or become?
**Interviewer's prompts throughout the interview**<br>"Can you tell me more about that?"<br>"How did that make you feel?"<br>"Can you give an example?"<br>"What was that experience like for you?"<br>"How did you and your family member experience that situation?"<br>"What meaning did you give to that experience?"	
